# Procalcitonin to stop antibiotics after cardiovascular surgery in a pediatric intensive care unit—The PROSACAB study

**DOI:** 10.1371/journal.pone.0220686

**Published:** 2019-09-18

**Authors:** Sara Bobillo-Perez, Anna Sole-Ribalta, Monica Balaguer, Elisabeth Esteban, Monica Girona-Alarcon, Lluisa Hernandez-Platero, Susana Segura, Aida Felipe, Francisco Jose Cambra, Cristian Launes, Iolanda Jordan

**Affiliations:** 1 Pediatric Intensive Care Unit, Hospital Sant Joan de Déu, Universitat de Barcelona, Barcelona, Spain; 2 Disorders of Immunity and Respiration of the Pediatric Critical Patients Research Group, Institut Recerca Hospital Sant Joan de Déu, Universitat de Barcelona, Barcelona, Spain; 3 Pediatrics Department, Hospital Sant Joan de Déu, Universitat de Barcelona, Barcelona, Spain; 4 Pediatric Infectious Diseases Research Group, Institut Recerca Hospital Sant Joan de Déu, CIBERESP, Barcelona, Spain; Vita Salute University of Milan, ITALY

## Abstract

**Introduction and objective:**

Children admitted to the pediatric intensive care unit after cardiovascular surgery usually require treatment with antibiotics due to suspicion of infection. The aim of this study was to assess the effectiveness of procalcitonin in decreasing the duration of antibiotic treatment in children after cardiovascular surgery.

**Methods:**

Prospective, interventional study carried out in a pediatric intensive care unit. Included patients under 18 years old admitted after cardiopulmonary bypass. Two groups were compared, depending on the implementation of the PCT-guided protocol to stop or de-escalate the antibiotic treatment (Group 1, 2011–2013 and group 2, 2014–2018). This new protocol was based on the decrease of the PCT value by 20% or 50% with respect to the maximum value of PCT. Primary endpoints were mortality, stewardship indication, duration of antibiotic treatment, and antibiotic-free days.

**Results:**

886 patients were recruited. There were 226 suspicions of infection (25.5%), and they were confirmed in 38 cases (16.8%). The global rate of infections was 4.3%. 102 patients received broad-spectrum antibiotic (4.7±1.7 days in group 1, 3.9±1 days in group 2 with *p* = 0.160). The rate of de-escalation was higher in group 2 (30/62, 48.4%) than in group 1 (24/92, 26.1%) with *p* = 0.004. A reduction of 1.1 days of antibiotic treatment (group 1, 7.7±2.2 and group 2, 6.7±2.2, with *p* = 0.005) and 2 more antibiotic free-days free in PICU in group 2 were observed (*p* = 0.001), without adverse outcomes.

**Conclusions:**

Procalcitonin-guided protocol for stewardship after cardiac surgery seems to be safe and useful to decrease the antibiotic exposure. This protocol could help to reduce the duration of broad-spectrum antibiotics and the duration of antibiotics in total, without developing complications or adverse effects.

## Introduction

The prescription and duration of antibiotic treatment have been carefully reviewed in recent years because of the growing incidence of infections and new emergent multidrug-resistant bacteria. Antimicrobial resistance is responsible for 35,000 deaths per year in Spain and 700,000 worldwide, and a progressive rise is foreseen, until reaching 10 million in 2050. In addition, healthcare-associated infections (HAI) affect 4.5 million patients worldwide, prolong length of stay (LOS) by 16 million days, and are responsible for 37,000 deaths each year in Europe. The annual cost of antimicrobial resistance and HAI in the European Union is around 1.5 and 7 billion euros, respectively. The maximum exponents of this situation are the intensive care units, where vulnerable critically ill patients require invasive devices and receive more antibiotics [1–3]. The extension of antimicrobial resistances, especially in the gram-negative bacilli, concerns all the medical community.

Post cardiovascular surgery (CVS) patients are a population that usually requires antibiotic treatment and HAI is a frequent complication. The incidence of HAI in these patients is about the 10–20% but interinstitutional variations exist [4–6]. Patients receive antibiotic prophylaxis before and after CVS. This prophylaxis is frequently changed by empirical antibiotic treatment when the patient develops signs and symptoms that are common for infection and systemic inflammatory response being difficult to distinguish both entities, especially in more complicated patients. The systemic inflammatory response is secondary to the released storm of cytokines after the extracorporeal circulation during CVS. This antibiotic treatment is usually indicated when patients have fever, hypotension, alteration of C-reactive protein, although most of the time, these symptoms are secondary to inflammation and not to infection. Once started, the duration of antibiotic treatment is mostly established by the clinical evolution and depending on the isolated microorganism in cultures. Recently, some biomarkers such as procalcitonin (PCT) have been added to the antibiotic treatment algorithms in order to give an objective parameter of bacterial infection and of the antibiotic response [7]. PCT is a well-known biomarker, mainly used for diagnosing bacterial infection in different settings, such as intensive care units. Its determination has been demonstrated to be helpful to differentiate post-surgery infection from systemic inflammatory response in adults and children [8–12], but also for guiding the antibiotic duration and improving antibiotic stewardship, especially in adults [13–18]. Studies regarding PCT-guided stewardship in children are limited.

Patients affected by complex heart disease may need more than one complex surgery in their childhood, with a long LOS in hospital. So limiting exposure to broad-spectrum antibiotics should be the goal of physicians in order to help in reducing multidrug resistance, to improve antibiotic strategies, to avoid secondary antibiotic effects, and to decrease costs.

The main aim of this study was to determine the effectiveness of PCT in decreasing the duration of antibiotic treatment in children after CVS. Secondary objectives were to determine if this approach based on a PCT-guided protocol helped to decrease pediatric intensive care (PICU) without increasing complications.

## Materials and methods

Prospective, unicenter, interventional study, conducted in a PICU. Inclusion criteria were children under 18 years of age who were admitted after CVS to the PICU of a third level hospital. Excluded criteria were newborn who were admitted to the neonatal intensive care unit after CVS (due to the different antibiotic policy of that unit), patients with rheumatologic disease (PCT value may increase in rheumatic outbreaks) or immunodeficiency (these patients receive individualised antibiotherapy), and patients with suspicion of community infections.

Two different groups of patients were compared over time. Group 1 included patients recruited from 2011 to 2013 and group 2 from 2014 to 2018. The difference between both groups was the implementation in group 2 of the PCT-guided protocol to stop or provide stewardship in the antibiotic treatment. Primary endpoints were death in the PICU, stewardship indication, duration of antibiotic treatment, and antibiotic-free days. Secondary endpoints were reinfections, relapses, and the LOS in PICU.

### Antibiotic policy

**Fig 1:**
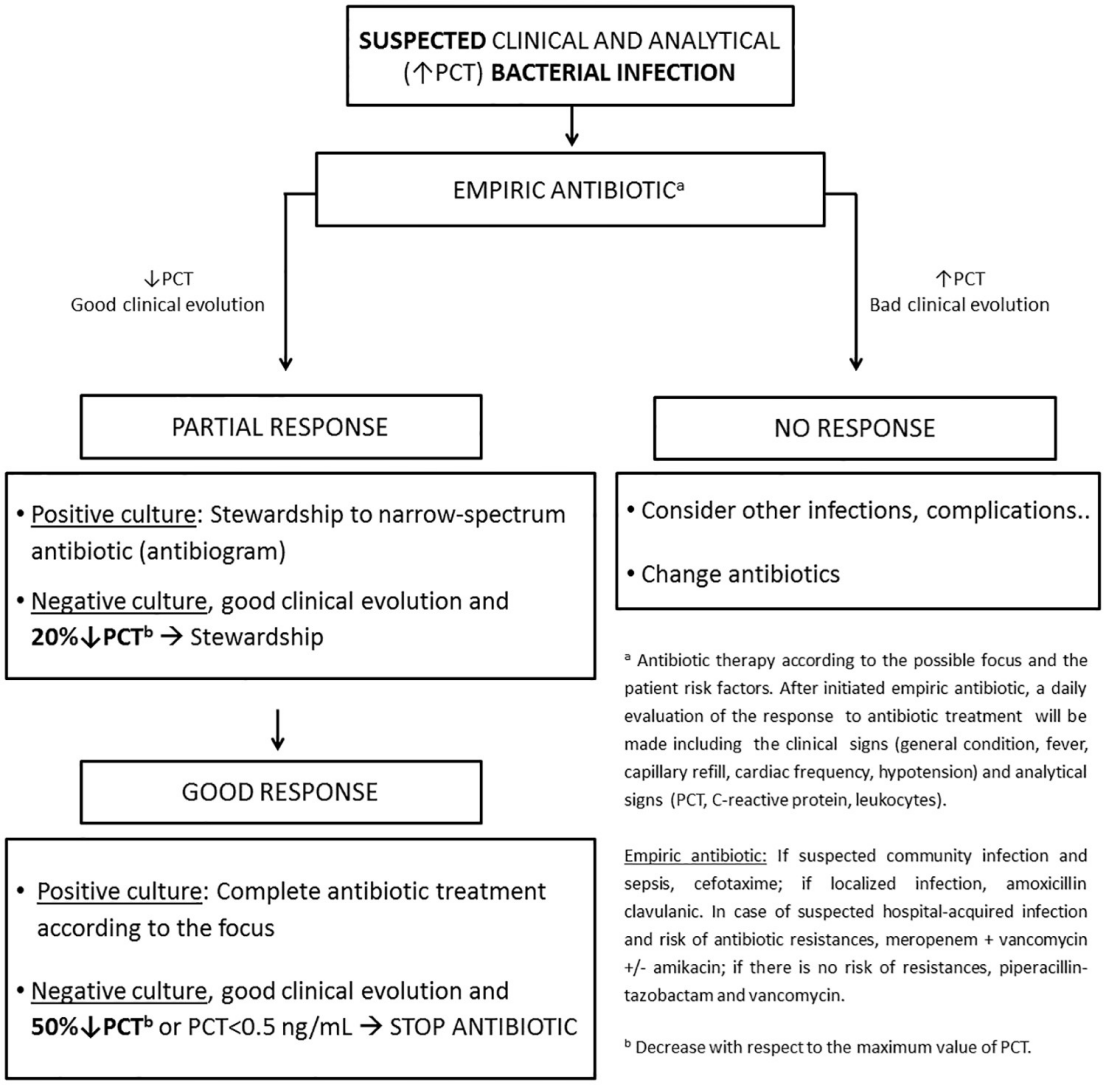
Algorithm of the procalcitonin-guided antimicrobial stewardship protocol. PCT: Procalcitonin.

After CVS all patients received antibiotic (cefazoline) as prophylaxis until the withdrawal of mediastinal drainage. Since 2010 the infection diagnosis and subsequent antibiotic treatment had been based on clinical (fever, bad general condition) and analytical (leukocyte count, C reactive protein, and PCT values [9]) data. The antibiotic duration was determined by the medical staff criteria, according to clinical evolution, culture results, and type of infection [19–21]. The stewardship to a narrow-spectrum antibiotic was recommended according to the laboratory sensitivity results. In 2014, a new antibiotic policy algorithm was defined for patients after CVS patients, based on an algorithm guided by PCT (Fig 1).

In relation to the suspension of the antibiotic, the new protocol added as a criterion the decrease in PCT values of more than 50% of the initial value or the decrease of the PCT value below 0.5 ng/mL. No other significant changes were introduced in the antibiotic policy algorithm. Nevertheless, the final decision is at the discretion of the attending physician.

For the study patients were diagnosed with nosocomial infection based on the Centre for Disease Control and Prevention criteria [22]. Sepsis diagnosis is defined according to the international definition of paediatric sepsis revised in 2012 [23]. Reinfection is considered as the development of a new infection after the first infection is solved. Relapse is defined as the development of an infection by the same bacteria that caused the first infection in the first two weeks after the previous one. De-escalation is considered as the change of the antibiotic to one of minor or narrow-spectrum. In group 1, the analysis was carried out by the researcher who, considering the medical evolution and results of the cultures, determined whether the de-escalation/stop would have been indicated. The indication of de-escalation/stop in group 2 was determined by the algorithm of the new protocol (Fig 1). The de-escalation indication includes those patients in which it is correctly applied and those in which, when indicated, it is not performed. De-escalation is not applicable when the initial therapy was correct for the confirmed bacterial infection or when the antibiotic was stopped early in the absence of signs of infection. The number of days of antibiotics includes prophylaxis and treatment. The antibiotic-free days are the difference between the duration of the antibiotics (including prophylaxis) and the LOS in PICU and in hospital.

PCT values were determined by LumiTest PCT immunoluminometric assay (ATOM SA; Brahms Diagnostica, Middletown, VA), which uses two monoclonal antibodies and requires 20 μL of serum or plasma. Serial PCT values were determined daily during the 3 first days after CVS and if a suspicion of infection existed, daily until the resolution of the process.

Baseline demographic data were recorded and included: type of congenital heart disease, complexity of surgery (Aristotle score [24]), surgery times (time of extracorporeal circulation, aortic cross clamp and deep hypothermic circulatory arrest), and Pediatric Risk Mortality Score (PRISM III). Haemodynamic and respiratory support in the first days after surgery were also analysed, both in absolute numbers, assessing the need for inotropes at 24 hours from surgery (different from milrinone, which per protocol is administered in the first hours after extracorporeal circulation) and intubation upon admission, such as the vasoactive-inotropic score (VIS) [25] and the hours of mechanical ventilation. Infection data included: device-related nosocomial infection (ventilator associated pneumonia, central line associated blood stream infection, catheter associated urinary tract infection), a diagnosis of sepsis, antibiotic treatment stewardship and duration, and antibiotic-free days. LOS in the PICU and in the hospital were recorded. Reinfection and relapse were considered as adverse outcomes.

SPSS 22.0 for Windows was used for statistical analysis. Qualitative variables were expressed as frequencies and percentages and compared using Pearson chi-square test. Quantitative variables were expressed by median and interquartile rang (IQR) and compared using Mann-Whitney test or mean and standard deviation depending on the distribution of the sample and compared using t-student test. Kruskall-Wallis test was also used for comparing various qualitative variables and a quantitative variable. In order to analyze the possibility of negative effects (increase in mortality or LOS) after the introduction of the new protocol, a multivariate analysis was proposed. A backward stepwise logistic regression was performed and results were expressed as Odds Ratio (OR) and confidence interval (CI95%). A *p*-value < 0.05 was considered significant.

The study was conducted in accordance with the Helsinki Declaration recommendations and approved by the local Ethical Assistance Committee (CEIm Fundacion Sant Joan de Deu, Barcelona) and the institutional review board with a waiver of individual informed consent.

## Results

A total of 886 patients were recruited, 371 in group 1 (41.8%, pre-implementation the PCT guided protocol) and 515 in group 2 (58.2%, post-implementation). The median age was 1.5 years (IQR 0.5–6.5) and 476 were males (53.7%). Two-hundred and seventy-four patients (30.9%) had previously undergone cardiac surgery and 142 patients (16%) had an underlying disease, 57 of them Down syndrome (40.1%). The main type of congenital heart disease was the left-to-right shunt heart defect, with 410 patients (46.3%); 282 patients (31.8%) had a cyanotic cardiac defect, 163 (18.4%) obstructive CHD, and, finally, 31 patients (3.5%) had other CHDs. The ventricular septal defects and the tetralogy of Fallot were the two most frequent CHDs with 152 (17.16%) and 133 patients (15%), respectively. Aristotle score was 2 in 410 patients (46.3%) and was higher than 2 in 257 patients (29%). The median PRISM III was 3 points (IQR 2–6). There were no statistically significant differences between the two groups according to any clinical data on previous surgery. All data are summarized in Table 1.

**Table 1:** General summary for the sample of the demographic data, surgical characteristics, and postoperative support in the paediatric intensive care unit.

Variables	TOTAL (*n* = 886)	GROUP 1 (*n* = 371)	GROUP 2 (*n* = 515)	*p*-value
		2011–2013	2014–2018	
**Demographic and surgery**				
Age (years)–Median (IQR)	1.5 (0.5–6.5)	1.6 (0.5–5.9)	1.4 (0.5–6.6)	0.816
Under 1 year–n, %	357 (40.3%)	143 (38.5%)	214 41.6%	0.368
Sex (males)–n, %	476 (53.7%)	206 55.5%	270 52.4%	0.361
Weight (Kg)–Median (IQR)	22 (10–51)	24 (11–55)	9 (21–48)	0.064
Previous cardiac surgery–n, %	274 30.9%	107 28.8%	167 32.4%	0.266
Underlying disease–n, %	142 16%	68 18.3%	74 14.4%	0.275
PRISM III score–Median (IQR)	3 (2–6)	3 (2–6)	3 (2–6)	0.553
Aristotle score (2 points)–n, %	410 46.3%	149 40.2%	261 50.7%	0.006
Aristotle score higher than 2– n, %	257 29%	144 38.8%	113 21.9%	0.281
EC–n, %	755 85.2%	338 91.1%	417 80.9%	0.617
Time of EC (min)–Median (IQR)	71 (50–90)	75 (54.8–97.3)	70 (46.25–98)	0.023
Time of clamp (min)–Median (IQR)	40 (25–62)	40 (28–61)	38 (23–62)	0.163
Time of DHCA (min)–Median (IQR)	27 (22–40)	27 (17.5–38.5	33 (21.5–41.25)	0.647
**Support after surgery**				
MV at admission–n, %	387 43.7%	155 41.8%	232 45%	0.289
Hours of MV–Median (IQR)	6 (3–24)	5 (3–24)	6 (3–24)	0.322
Inotropics in PICU*– n, %	183 20.7%	66 17.8%	117 22.7%	0.074
VIS at 24 hours–Median (IQR)	3.7 (3.7–7)	3.7 (3.7–3.7)	3.7 (3.7–7)	<0.001
VIS at 48 hours–Median (IQR)	3.7 (0–3.7)	3.7 (0–3.7)	0 (0–3.7)	0.016
Need for re-intervention**– n, %	25 2.8%	6 1.6%	19 3.7%	0.155
Need for RRT–n, %	13 1.5%	4 1.1%	9 1.7%	0.414
Need for ECMO–n, %	8 0.9%	2 0.5%	6 1.2%	0.331
**Outcomes**				
LOS in PICU (days)–Median (IQR)	3 (2–5)	3 (2–5)	3 (2–6)	0.120
LOS in hospital (days)–Median (IQR)	7 (6–10)	7 (6–9)	7 (6–10)	0.207
Exitus in PICU–n, %	11 1.2%	6 1.6%	5 1%	0.391

Qualitative values expressed by frequencies (Percentages) and compared with Pearson’ Chi-square test. Quantitative values expressed by median (INTERQUARTIL RANGE).

Mann-Whitney test.

*Need for inotropic support in PICU different from milrinone.

** Including urgent cardiac catheterization or cardiovascular surgery.

AB: Antibiotic; EC: Extracorporeal circulation; CHD: Congenital heart disease; DHCA: Deep hypothermic circulatory arrest; ECMO: Extracorporeal membrane oxygenation therapy; LOS: Length of stay; min: minutes; MV: Mechanical ventilation; PICU: Paediatric Intensive Care Unit; PRISM: Paediatric risk mortality score; RRT: Renal replacement therapy; VIS: vasoactive-inotropic score.

There were no differences according to need for haemodynamic and respiratory support, renal replacement therapy, or ECMO. Patients in group 2 needed more haemodynamic support in the first 24 hours but not in the 48 hours after surgery. Twenty-five (2.8%) needed an urgent re-intervention, in 5 cases due to severe bleeding. The median number of days of antibiotic was 2 (IQR 2–4). The prescription of antibiotics was greater in group 1 (113 patients, 30.5%) than in group 2 (114 patients, 22.1%) with *p* = 0.004.

**Fig 2:**
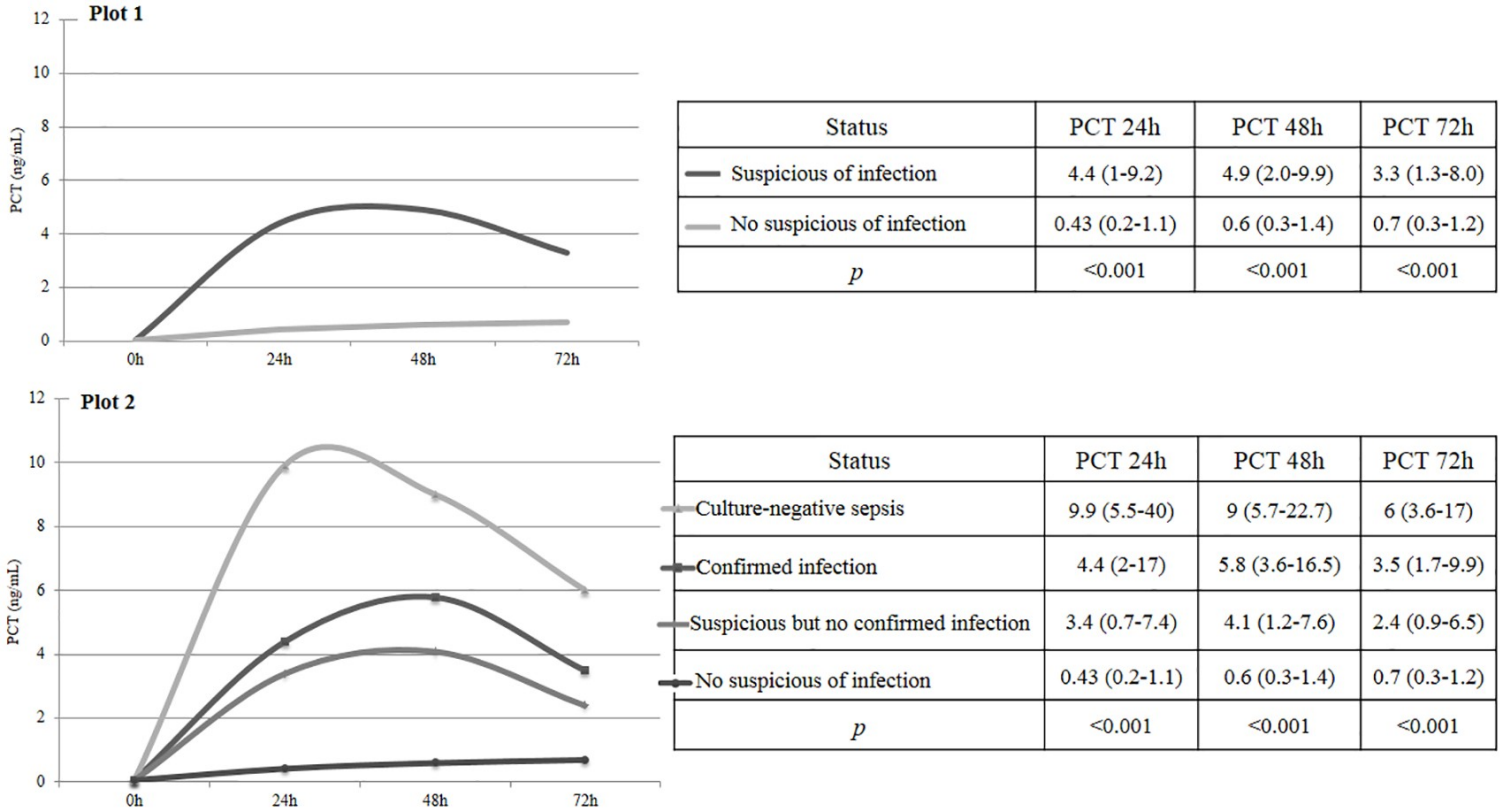
Graphic of the evolution of procalcitonin (PCT, ng/mL) over time according to the confirmation of infection in patients with suspicion of infection (all together). Mann-Whitney U test.

There were 226 patients with suspicion of infection (25.6%), 114 (30.7%) in group 1 and 112 (21.7%) in group 2 (*p* = 0.002). The infection was confirmed in 38 patients (38/226, 16.8%) and the global rate of infection was 38/886 (4.3%). In patients with suspicion of infection, the confirmation of it was greater in group 1, but without statistically significant differences (group 1, 22 patients, 19.3% *vs* group 2, 16 patients, 14.3%, *p* = 0.314). The evolution of PCT over time according to the confirmation of infection in patients with suspicion of infection is represented in Fig 2.

**Fig 3:**
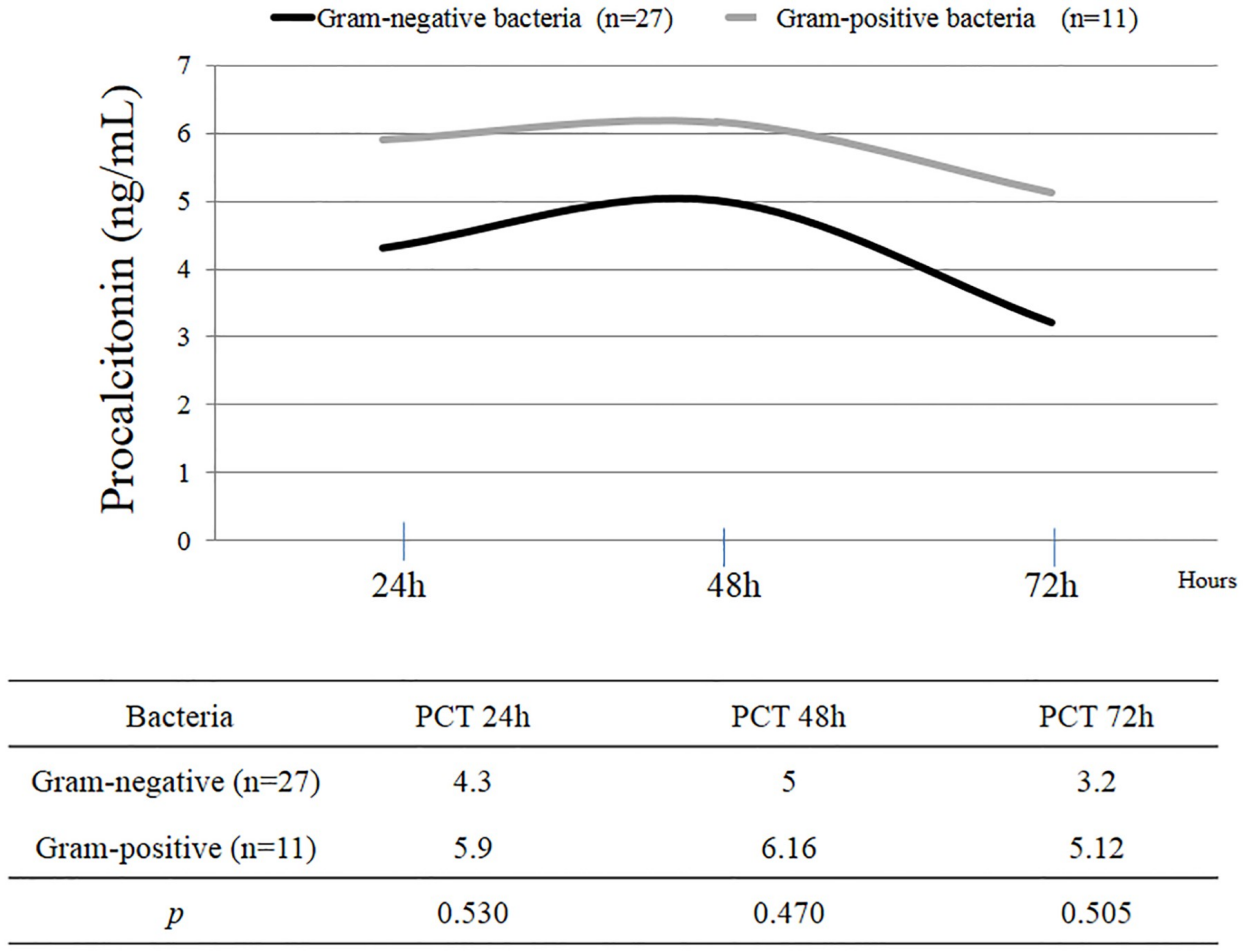
Representation of the evolution of procalcitonin (PCT, ng/mL) in infected patients according to the type of microorganism. Mann-Whitney U test.

The median value for PCT at the beginning of the empiric antibiotic treatment was 5.9 ng/mL (IQR 2.6–13.6), being higher in those in whom the infection was finally confirmed (6.7 ng/mL, IQR 4–38.6), vs 5.05 (IQR 2–9.9) than those without confirmed infection (p = 0.03)). The median value of PCT at the stewardship (stop or de-escalation) was 0.53ng/mL (IQR 0.2–2.77). The most frequent infections were ventilator associated pneumonia (30, 13.3%), followed by sepsis (6, 2.7%); Culture-negative sepsis were more frequent in group 2 with statistically significant differences (*p* = 0.036). Table 2 summarizes the differences between the two groups and each type of infection. Gram-negative bacteria were the most frequent type of isolated microorganism (27 patients), followed by gram-positive bacteria (11 patients). Fig 3 represents the evolution of PCT in patients infected by each type of microorganism. Patients infected by gram-positive bacteria tended to have higher PCT values but no statistically significant differences were detected (at 24, 48 and 72 hours, p = 0.530; 0.847 and 0.505 respectively).

**Table 2:** Summary of the patients with suspicion of infection.

Variables	TOTAL (*n* = 226)	GROUP 1 (n = 114)	GROUP 2 (n = 112)	*p*-value
		2011–2013	2014–2018	
**Broad-spectrum AB**–n, %	102, 45.1%	51, 44.7%	51, 45.5%	0.282
**Confirmed bacterial infection**–n, %	38, 16.8%	22, 19.3%	16, 14.3%	0.314
**Infection site**–n, %				
VAP	30, 13.3%	17, 14.9%	13, 11.6%	0.464
CLABSI	6, 2.7%	4, 3.5%	2, 1.8%	0.420
CAUTI	1, 0.4%	0, 0%	1, 0.9%	0.312
Wound	1, 13.2%	1, 0.9%	0, 0%	0.321
**Probable infection**: **Culture-negative sepsis**–n, %	30, 13.3%	7, 6.1%	23, 20.5%	0.036
**AB de-escalation**–n, %				
Yes	54, 23.8%	24, 21.1%	30, 26.7%	0.260
Not applicable	73, 32.2%	22, 19.5%	51, 44.7%	<0.001
No stop/No de-escalation	100, 44.1%	68, 60.2%	32, 28.1%	<0.001
**Days to stewardship**				
Mean± SD	4±1.4	4.47±1.7	3.7±1.2	0.100
Median (IQR)	4 (3–5)	4 (3–5)	3 (3–5)	0.173
Total days of AB*				
Mean± SD	7.21±0.16	7.7±2.2	6.7±2.2	0.005
Median (IQR)	7 (6–8)	7 (6–9)	7 (5–8)	0.003
**AB-free days in PICU**				
Mean± SD	2.33±11.5	0.17±6.8	4.5±14.4	0.005
Median (IQR)	-1 (-3-2)	-2 (-3-1)	0 (-2-4)	0.001
**AB-free-days in total**				
Mean± SD	9.7±18.6	6.8±11.9	12.5±23.2	0.023
Median (IQR)	4 (1–9)	3 (0–8)	5 (1–13)	0.015
**Reinfection**–n, %	24 (10.6%)	10 (8.8%)	14 (12.5%)	0.375
**Relapse**–n, %	0	0	0	
**LOS in PICU (days)**				
Mean± SD	9.5±11.9	7.8±8	11.1±14.7	0.037
Median (IQR)	6 (3–9)	6 (3–9)	6 (4–12.8)	0.179
**Exitus in PICU**–n, %	7 (3.1%)	5 (4.4%)	2 (1.8%)	0.259

Qualitative values expressed by frequencies (Percentages) and compared with Pearson’ Chi-square test. Quantitative values expressed by mean (STANDARD DEVIATION, SD), compared with student-t test, and also expressed by median (INTERQUARTIL RANGE, IQR) and compared with Mann-Whitney Test.

*The duration of antibiotic includes the days of prophylaxis and treatment.

AB: Antibiotic; CAUTI: catheter-associated urinary tract infections; CLABSI: central-line associated bloodstream infections; LOS: Length of stay; min: minutes; PICU: Paediatric Intensive Care Unit; VAP: ventilator-associated pneumonia.

The main isolated bacteria were *Pseudomona aeruginosa* and *Enterobacter cloacae*, with 5 patients each. There were 4 positive cultures for *Klebsiella pneumoniae* and 4 for *Serratia marcescens*. Three patients were infected by *Candida* spp. No multidrug-resistant bacteria were isolated. There were no statistically significant differences in the use of broad-spectrum antibiotics between the two groups. All data are summarized in Table 2.

The de-escalation would have been indicated in 154 patients, 92 in group 1 (81.4%) and 62 in group 2 (54.8%), with *p<0*.*001*, but it was only done in 24 patients in group 1 (24/92, 26.1%) and in 30 patients in group 2 (30/62, 48.4%). Differences were statistically significant with a higher rate of de-escalation in group 2 than in group 1 (*p* = 0.004). There were no differences regarding the de-escalation to an antibiotic of minor spectrum: 24/114 (21.1%) in group 1 *vs* 30/112 (26.7%) in group 2 with *p* = 0.260. However, the de-escalation was not applicable in more patients in group 2 (51/114, 44.7%) than in group 1, (22/113, 19.5%) with *p<0*.*001*. On the other hand there were fewer de-escalations or early cessations of the antibiotic in group 2 (group 1, 68/113, 60.2% vs group 2, 32/114, 28.1%, with *p<0*.*001*). The de-escalation was performed before in group 2 but statistically significant differences were not demonstrated (group 1, 4 days until de-escalation (IQR 3–5) vs group 2, 3 days (IQR 3–5) with *p* = 0.173). In those patients who received broad-spectrum antibiotics (*n* = 104), there were differences according to the number of days of broad-spectrum antibiotic treatment before de-escalation of the treatment, with a reduction of 0.7 days of broad spectrum antibiotic (group 1, 4.7±1.7 days vs group 2, 3.9±1 days with *p* = 0.160).

There were statistically significant differences between the two groups according to the duration of antibiotic: the median number days of antibiotics was 7 (IQR 6–9) in group 1 and 7 (IQR 5–8) in group 2, with p<0.001and a significant reduction of 1.1 days (mean in group 1, 7.7±2.3 and mean in group 2, 6.6±2.4, with *p*<0.005). The antibiotic-free days in PICU were different in the two groups, with 2 more days free of antibiotics in PICU in group 2 than in group 1 (*p*<0.005). According to the antibiotic-free days during hospitalization, there were also 2 more days free of antibiotics in group 2, with *p* = 0.009. All data are summarized in Table 2. In the overall sample (*n* = 886), no differences according to mortality in PICU were observed (6 in group 1, 1.6%; and 5 in group 2, 1%; *p* = 0.391). Likewise, in suspected infected patients (*n* = 227), no differences in mortality were detected (group 1, 5, 4.4% vs group 2, 2 patients, 1.8%, *p* = 0.245). One patient from the group 1 died due to an infectious disease with shock: the bloodstream culture was positive for *Staphylococcus epidermidis* 24 hours after surgery.

No differences were observed in the reinfection rate in the entire sample (group 1, 13/371 patients and 3.5%; group 2, 21/515 patients and 4.1%, *p* = 0.667). Relapse was not detected. There were no differences in LOS in PICU (group 1, 3 days (IQR 2–5) and group 2, 3 days (IQR 2–6), with *p* = 0.120) and LOS in hospital (group 1, 7 days (IQR 6–9), group 2, 7 days (IQR 6–10). There were also no statistically significant differences when the LOS was analysed only in patients with suspicion of infection (227): in PICU group 1, 6 days (IQR 3–9) and group 2, 6 days (IQR 4–12.8), with p = 0.179; and in hospital, group 1, 9 days (IQR 8–15.5) and group 2, 11 days (IQR 8–21) with p = 0.316. In the multivariate analysis, age under 1 year (OR 2.786, CI95% 1.181–6.572, *p* = 0.019) and hours of MV (OR 1.015, CI95% 1.004–1.025, *p* = 0.005) were independently associated with a prolonged LOS in PICU (more than 5 days) in patients with suspicion of infection. The prolonged LOS in the PICU was not related to the protocol group, the confirmation of infection, the inotropic score at 24 hours, nor to the Aristotle score higher than 2, the times of surgery, the need for renal replacement therapy or ECMO. The reinfection entailed an increase in the number of days in PICU and hospital in both groups (group 1 the median days in PICU with reinfection was 11 (IQR 7–18.5) and without reinfection, 3 days (IQR 2–5); in group 2, the median days in PICU with reinfection was 16 (IQR 4.5–32) and without reinfection, 3 (IQR 2–5). The LOS in hospital was also larger: in group 1, the median days in case of reinfection was 23 (IQR 14–34.5) and without reinfection, 7 (IQR 6–9); in group 2, with reinfection the median days was 30 (13–43.5) and without reinfection, 7 days (IQR 6–9). *p* value was <0.001 in all cases.

## Discussion

To our knowledge, this is the first study in children in which the implementation of a PCT-guided protocol for antibiotic stewardship after CVS has been evaluated. Results have shown an improvement in the antibiotic policy, with a reduction of 8.4% in the utilization of antibiotics and a greater awareness of the need to optimize their use. The decision of stewardship was similar in the two periods, but the stewardship was 1 day earlier in group 2. Also stewardship was not applicable to more patients in group 2 because of the optimal empiric treatment that was initiated. The antibiotic duration was also 1 day less in the PCT-guided group and there were 2 more antibiotic-free days.

Concern about the policy of antibiotic exposure is high due to the rise in emerging multi-resistant bacteria. This increase is especially related to the broad-spectrum antibiotic indication but even the duration of prophylactic antibiotic is important in order to prevent new resistance [26]. PCT has shown its usefulness in differentiating bacterial infection from inflammation after CVS, which allows avoiding the indication of antibiotics [9,11]. After CVS, patients may be very unstable, and broad-spectrum antibiotics are commonly started due to a possible infectious trigger, despite the laboratory results. Therefore, biomarkers could help in a second step, in order to improve antibiotic stewardship and reduce the duration of the antibiotics.

Most data on antibiotic stewardship guided by PCT are published about adults, among whom the rise of multi-resistances is a real and important problem nowadays. One of the first studies was published in 2010 (PRORATA trial)[16], a multicentre randomised and controlled trial in adults admitted to an intensive care unit. In this study, the group that followed a protocol guided by PCT received 2.7 fewer days of antibiotics than the control group, without adverse effects. Similar studies were later published [27], and two recent meta-analyses confirmed the usefulness of PCT for reducing antibiotic exposure of adults in intensive care units without an increase in complications [18,28]. However, the authors noted two different types of pathologies in which the duration of the antibiotic treatment was not modified by the PCT-guided protocol: abdominal infections and renal impartment [18]. In addition, a recent Cochrane review concluded that current data do not provide good evidence to support PCT-guided protocols, possibly due to the lack of homogeneity among the included studies [29]. A 2017 Cochrane review in adults recommended the use of PCT for guiding the cessation of antibiotics in acute respiratory infection because of the reduction in both the number of days with antibiotics and in mortality [30]. In contrast with these results, a recent study in adults with suspected low-respiratory tract infection did not yield evidence of a reduction in the use of antibiotics with a PCT-guided protocol [31]. Probably this was secondary to low adherence to the protocol and also because a high rate of patients in whom bacterial infection would not be suspected using clinical signs were included, thereby underestimating the usefulness of PCT-guided protocols in pathologies with which physicians would use antibiotics more consistently. In surgery patients, some authors have demonstrated the usefulness of PCT guided protocols, as in peritonitis cases, with a 50% reduction in antibiotic duration [32]. There are no data in adults about the use of a PCT-guided protocol in patients after CVS.

Data in PICU population are scarce. In newborns with early-onset sepsis, PCT-guided antibiotic protocol showed a reduction of 22.4 hours of antibiotics [14]. There was also demonstrated to be a decrease in the exposure to antibiotics in children with nosocomial infection in PICU [33] with a reduction of 2 days of antibiotics following a PCT-guided protocol similar to the one used in our study. No increase in complications or adverse outcomes was detected. There are no published studies about the use of PCT-guided protocols in children after CVS, although preliminary published results seem to indicate that the use of PCT could reduce the exposure to antibiotics in these patients [34].

In the present study, the local antibiotic policy algorithm in these patients was changed due to the concern of the abuse of antibiotics. Apart from the introduction of the PCT criteria, no other significant changes were made in the protocol due to the very low impact of multidrug-resistant bacteria in our unit. Our results shown that there was not an increase in the stewardship after the implementation of the PCT-guided protocol. However, there was a trend toward reduction of the exposure to broad-spectrum antibiotics (1 day less) and also a day less of antibiotics, which meant 2 more antibiotic-free days in the PICU and the hospital. These results can have a great long-term impact, both at the clinical level, helping to reduce multi-resistance, and at the financial level, by saving antibiotics. One of the concerns about the reduction of the duration of antibiotic therapy is the upturn in reinfections and relapse. In our data, there was no variation between cohorts, confirming the safety of the newly implemented strategy. Previous data of a similar nature also showed no increase in adverse events [14,33,35]. During the study, our local protocol included the prophylaxis until the remove of the mediastinal drainage (usually 48 hours, as the previous recommendations of The Society of Thoracic Surgeons [36]). However, due to concerns about antimicrobial resistances, our current prophylaxis policy has changed after the end of this study to 24 hours [37].

Our secondary objective was to determine if the new protocol helped to reduce the number of days in PICU. However it was found an increase of LOS in PICU in the group guided by PCT. The multivariate analysis did not show that this was due to the new protocol, but factors such as age under 1 year or hours of MV were probably the factors that conditioned this increase in LOS.

This study presents some limitations because it is a pilot study. One important limitation is the single-center design, but on the other hand, a large sample of subjects was included. Another limitation is the exclusion of the patients from the neonatal intensive care unit. Cardiovascular surgeries in the neonatal period are more complex than in infants and children. Newborns usually require higher respiratory and inotropic supports and usually have an increased risk of infection. However, because these are two different units (PICU and the neonatal unit) with different antibiotic policies, patients from the neonatal intensive care unit were excluded in our analysis. Another important limitation is the fact that group 1 and 2 were recruited at different time periods, making it more difficult to draw binding conclusions. However, results are promising as a preliminary study. In the future, a multicenter study or even a clinical trial would be interesting in order to confirm the present results.

## Conclusions

PCT-guided protocol for stewardship in children after cardiac surgery seems to be safe and useful to decrease the antibiotic exposure. This protocol could help to reduce the number of days on broad-spectrum antibiotics and the number of days on antibiotics in total, without risk of developing complications or adverse effects. Resistance is combatted effectively with an optimal antibiotic policy. However, more research is needed to extend our knowledge of this biomarker and its usefulness in this kind of protocol, and pediatric clinical trials would be useful.
